# Clinical Paradox: Anterior Wall Myocardial Infarction with Predominant Inferior ST Elevation and No Variation in Coronary Anatomy in a PAI-1 Homozygote

**DOI:** 10.1155/2020/4172050

**Published:** 2020-10-06

**Authors:** Marko Perčić, Tea Friščić, Jasna Čerkez Habek, Dean Strinić, Ninoslav Rudman, Jozica Šikić

**Affiliations:** ^1^Department of Cardiology, Clinic of Internal Medicine, “Sveti Duh” University Hospital, Zagreb, Croatia; ^2^Department of Radiology, “Sveti Duh” University Hospital, Zagreb, Croatia

## Abstract

Changes of the ST segment are commonly used as predictors of the culprit vessel during an acute myocardial infarction. In case of combined ST elevation in both inferior and anterior leads, these changes can be due to a distal occlusion of a “wrapped” left anterior descending artery (LAD) or a two-vessel disease. Our case of anterior wall myocardial infarction with inferior ST elevation and anterior ST depression shows that electrocardiographic changes during acute myocardial infarction cannot always be explained by logical sequelae of the injury current, vessel anatomy, and their irrigation territory.

## 1. Introduction

ST segment changes, whether elevation or depression, as well as their distribution are commonly used as predictors of the culprit vessel during an acute myocardial infarction. ST elevation in inferior leads suggests an acute occlusion of the right coronary artery (RCA) or the left circumflex coronary artery (LCx). In case of combined ST elevation in both inferior and anterior leads, these changes can be due to a distal occlusion of a “wrapped” left anterior descending artery (LAD) or a two-vessel disease [1]. There are several studies and case reports presenting the “wrapped” LAD being the cause of ST elevation in inferior and anterior leads, defining the “wrapped” LAD as a LAD from a postreperfusion coronary angiogram that perfuse at least one-fourth of the inferior wall of the left ventricle in the right anterior oblique projection [2–8]. According to a recent study, hereditary thrombophilia was the most frequently encountered atypical etiology for acute myocardial infarction in young people, accounting for 15% of cases [9]. We report a rare case of ST elevation in inferior leads following proximal LAD occlusion without coronary anatomy deviation and subsequent anterior wall myocardial infarction in a patient with PAI-1 mutation.

## 2. Case Presentation

**Figure 1:**
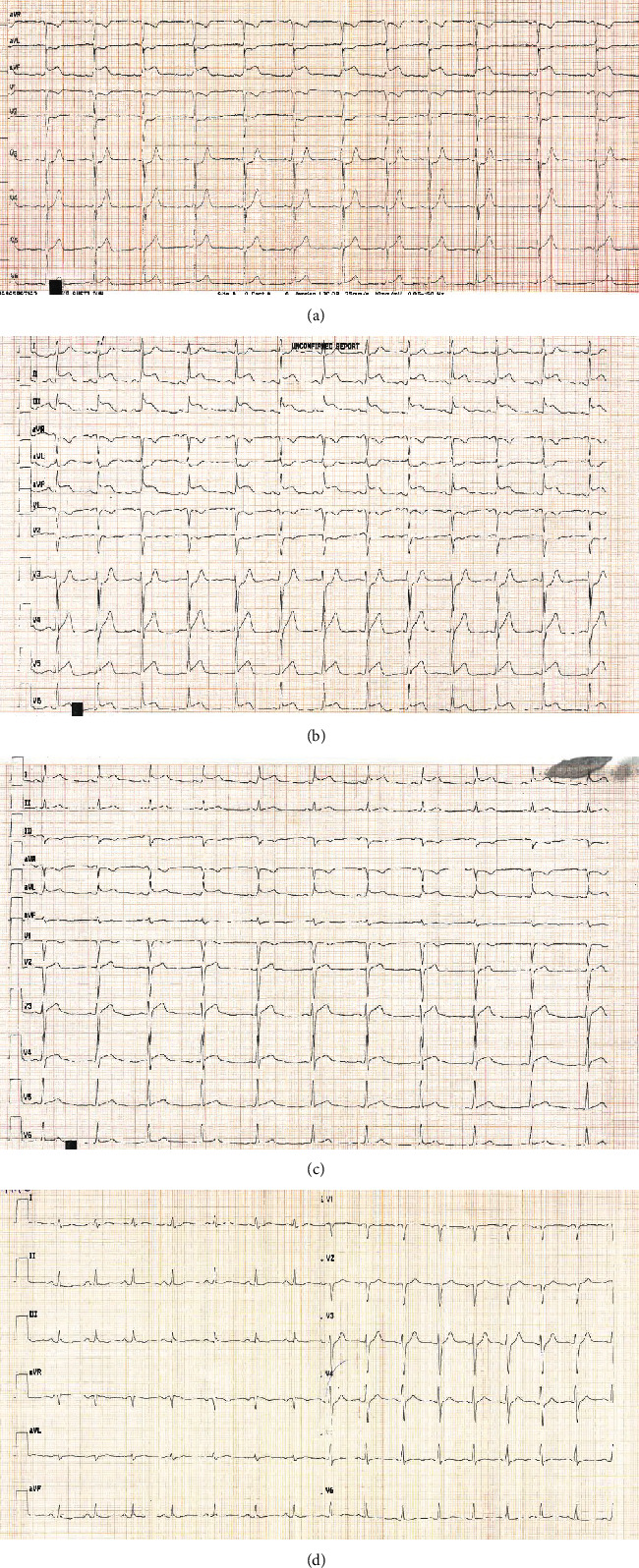
(a) ECG in the emergency department. (b) ECG at the admission to the ICU. (c) ECG taken 12 h after pPCI. (d) ECG taken 4 months after pPCI.

**Figure 2:**
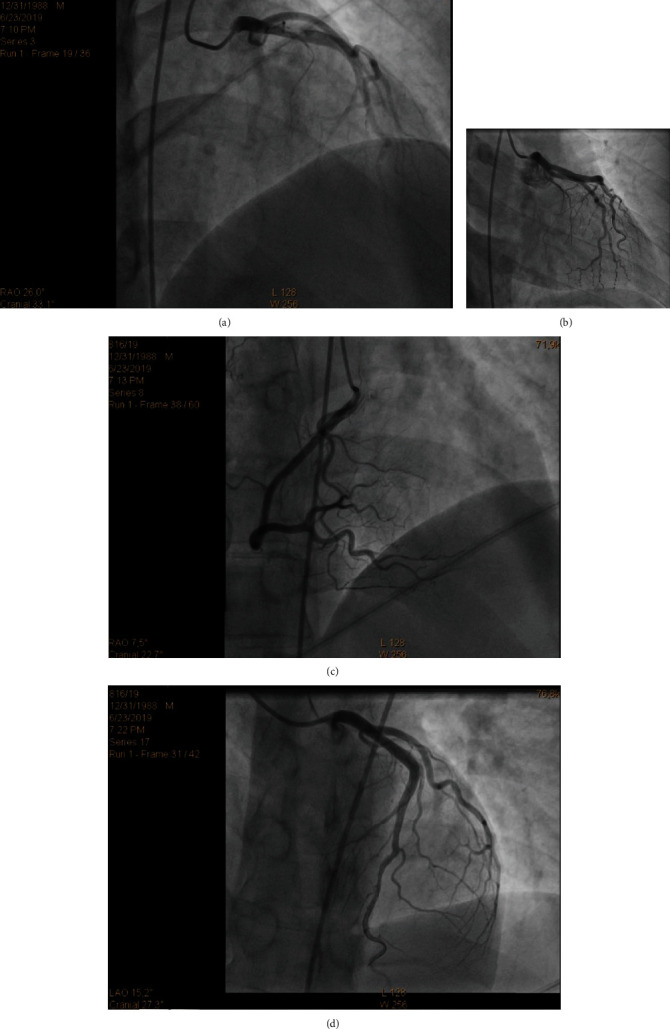
(a) Left anterior descending artery—proximal segment subocclusion. (b) Left anterior descending artery—RAO. (c) Right coronary artery. (d) Left anterior descending artery after pPCI with drug eluting stent (4 × 15mm).

**Figure 3:**
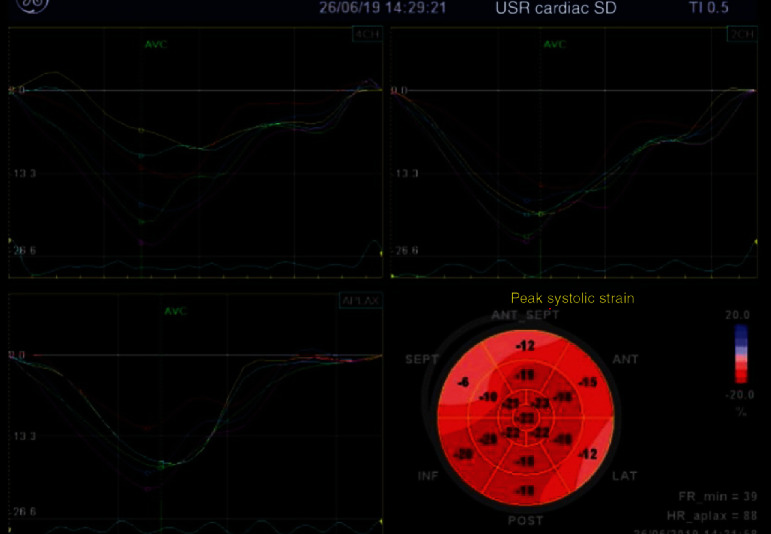
Peak longitudinal strain 3 days after myocardial infarction showing reduced strain in basal anteroseptolateral segments while inferior and posterior segment remained intact.

**Figure 4:**
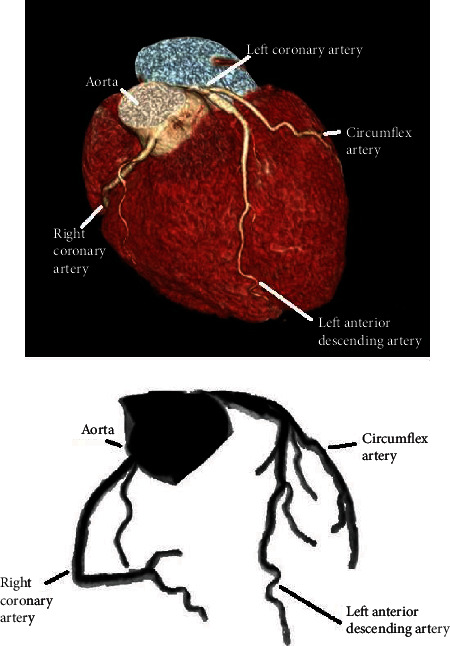
MSCT coronary angiography showed no signs of stenosis and no variations in coronary anatomy.

A 30-year-old male patient with no previous history of cardiovascular disease, cardiovascular risk factors, or family history of cardiovascular disease presented in emergency department with retrosternal chest pain not related to exertion that had started four hours before. There was no pain radiation, nausea, or vomiting. He had no recent history of infectious disease (as a pericarditis/myocarditis trigger). On physical examination, there were no abnormal findings. The initial electrocardiogram (ECG) showed 2 mm ST segment elevation in leads II, III, and aVF; 1 mm in V6; and horizontal ST depression in aVL and V1-3 (Figure 1(a)). His blood pressure was 180/105 mmHg, his heart rate was 65/minute, and he was afebrile. The patient was administered acetylsalicylic acid 300 mg and ticagrelor 180 mg orally and then admitted to coronary intensive care unit (ICU) where the second ECG showed more prominent ST elevation in leads II, III, aVF, and V5-6 and ST denivelation in aVL and V1-3 (Figure 1(b)). Initial troponin on presentation was 41 ng/L (referent value < 40 ng/L). He was taken up for coronary angiography immediately after admission to the ICU. Coronary angiogram showed right-dominant system (Figure 2). LAD had a microathero/thrombotic subocclusion of the proximal segment with initial TIMI 2 flow and a small embolus in the distal segment. RCA showed slow flow phenomenon with no signs of stenosis. Primary percutaneous coronary intervention (pPCI) was preformed, and one drug-eluting stent (4.0 × 15mm) in LAD has been implanted. ECG taken 12 hours after pPCI showed 1.5 mm ST elevation in leads I and aVL and formed Q with inversed T wave in lead III (Figure 1(c)). The laboratory test showed LDL levels of 2.85 mmol/L and total cholesterol of 4.79 mmol/L. All the other results were within normal range, including electrolyte levels, and the peak troponin level was 22845 ng/L. There were no postprocedural complication, and the patient had stable hemodynamics and rhythm until discharge. The therapy he received was according to the latest European guidelines for the treatment of acute coronary syndrome. Three days after pPCI, echocardiographic global longitudinal strain was -13.8% with reduced strain in basal anteroseptolateral segments of the left ventricle, while inferior and posterior segments remained intact (Figure 3). Three months after myocardial infarction, multislice computed tomography (MSCT) coronary angiography was performed, showing no variations in coronary anatomy (including “wrapped” LAD), no abnormal heart rotation, and no signs of stenosis (Figure 4). Thrombophilia testing was also performed, and the results came in negative for factor V Leiden (R506Q) and factor II prothrombin (G20210A), with no antithrombin III or protein S deficiency, or increase in lupus anticoagulant, MTHFR (C677T) heterozygote, and PAI-1 (4G/5G) homozygote 4G. Patient has been asymptomatic since the discharge and is on regular follow-up. ECG taken 4 months after the acute myocardial infarction shows Q in V1-2, with no Q in inferior leads.

## 3. Discussion

During acute anterior wall myocardial infarction, elevation of the ST segment is usually seen in the anterior leads. ST segment depression is sometimes observed simultaneously in the inferior leads which is generally considered to be a reciprocal change of ST elevation in aVL related to anterolateral extension of the ischaemia of D1 branch irrigation territory [7].

Inferior ST elevation during acute anterior myocardial infarction is unusual but explainable when the “wrapped” LAD is occluded distal to its first diagonal branch, appearing with a combination of a transmural ischaemic myocardium in the anterobasal and anterolateral wall of a lesser degree together with transmural ischaemic myocardium in the inferior wall [10].

Sapin et al. explained the ST segment changes in “anterior-inferior” myocardial infarction with the distal occlusion in the LAD which allows the more proximal patent branch vessels to perfuse the anterior wall resulting in a smaller mass of ischemic anterior wall myocardium, thus smaller anterior wall injury current and less reciprocal inferior ST segment depression. Inferior wall injury current, as a result of occluded “wrapped” LAD, must be combined with the weak reciprocal of a smaller anterior injury current to generate the ST elevation in the inferior leads [4]. Sasaki et al. had drawn a similar conclusion [8].

Our case describes a rare form of anterior myocardial infarction with inferior ST elevation with no deviation in coronary anatomy and right-dominant system. Also, there was no inferior wall myocardial infarction and no ST elevation in anterior leads. The patient is a PAI-1 4G homozygote, which some authors consider as an independent risk factor for acute myocardial infarction in young patients [11]. To our knowledge, there is no relationship between PAI-1, patient age, and ECG changes. We stressed the age of the patient and PAI-1 because the reason for LAD subocclusion was probably embolism and not a rupture of atherosclerotic plaque. These changes of the ST segment are hard to explain, especially with the results of the peak systolic strain which shows no reduction in inferior segments and a significant strain reduction in anterior segments. Also, in the ECG taken 4 months after pPCI, there was Q in septal leads. One of the possible explanations could be a spontaneous recanalization of the RCA.

## 4. Conclusion

Inferior ST elevation in an acute myocardial infarction of anterior wall is usually combined with anterior ST elevation. Both of the electrocardiographic changes are caused by occlusion of a “wrapped” LAD distal to D1 branch. Our case of anterior wall myocardial infarction with inferior ST elevation and anterior ST depression shows that electrocardiographic changes during acute myocardial infarction cannot always be explained by logical sequelae of the injury current, vessel anatomy, and their irrigation territory.
